# Continuous Alterations in the Gut Microbial Landscape Associated With Suicidal Ideation in First‐Episode Drug‐naïve Major Depressive Disorder

**DOI:** 10.1002/cns.70892

**Published:** 2026-06-18

**Authors:** Liqin Liang, Shuhao Chen, Baoyuan Zhu, Yuanyuan Huang, Xiaodan Lu, Shuhong Wang, Wei Wang, Rui Han, Jing Zhou, Dongsheng Xiong, Hehua Li, Xiaobo Li, Yuping Ning, Fengchun Wu, Kai Wu

**Affiliations:** ^1^ School of Biomedical Sciences and Engineering South China University of Technology, Guangzhou International Campus Guangzhou China; ^2^ School of Materials Science and Engineering South China University of Technology Guangzhou China; ^3^ Department of Psychiatry, the Affiliated Brain Hospital Guangzhou Medical University Guangzhou China; ^4^ Guangdong Engineering Technology Research Center for Translational Medicine of Mental Disorders Guangzhou China; ^5^ National Engineering Research Center for Tissue Restoration and Reconstruction South China University of Technology Guangzhou China; ^6^ Department of Biomedical Engineering New Jersey Institute of Technology Newark New Jersey USA; ^7^ Key Laboratory of Neurogenetics and Channelopathies of Guangdong Province and the Ministry of Education of China Guangzhou Medical University Guangzhou China; ^8^ Department of Aging Research and Geriatric Medicine, Institute of Development, Aging and Cancer Tohoku University Sendai Japan

**Keywords:** clinical association, gut microbiome, major depressive disorder, suicidal ideation

## Abstract

**Background:**

Major depressive disorder (MDD) has been closely associated with gut microbiota dysbiosis; however, the role of the gut microbiota in the progression from depression to suicidal ideation (SI) remains unclear.

**Methods:**

We enrolled a well‐characterized clinical cohort of first‐episode, drug‐naïve MDD patients, explicitly classified into those without SI (MDDNSI) and those with SI (MDDSI), and matched with healthy controls (HC) on demographic variables. A severity‐ordered HC‐MDDNSI‐MDDSI framework was established to capture progressive microbial and functional shifts, and correlation analyses were used to evaluate their relationships with clinical symptoms and cognitive function.

**Results:**

We identified a group of taxa showing clear severity‐related trends, with the potential pathogenic species *Bacteroides stercoris* and *Bacteroides eggerthii* increasing across the clinical spectrum, while seven species, including *Faecalibacillus intestinalis* and *Dialister massiliensis,* showed a steady decrease. Functional annotation indicated that several major metabolic pathways, such as the bacterial secretion system, weakened progressively with disease severity and formed stable microbe‐pathway modules together with pathways involved in energy metabolism and signal transduction. These differential taxa and pathways showed strong associations with clinical features, with *Bacteroides stercoris* positively correlated with SI, whereas *Bifidobacterium pseudocatenulatum* displayed a negative association. In addition, mediation analysis further showed that *Bacteroides stercoris* indirectly influenced SI through the bacterial secretion system pathway, suggesting a meaningful mediating role in SI.

**Conclusion:**

These results revealed progressive alterations in gut microbial composition and metabolic function associated with SI, indicating that gut dysbiosis serves as a potential biological marker for suicide risk in MDD.

## Graphical abstract

We define a gut‐microbiota gradient linked to increasing depression severity and suicidal ideation, highlighting severity‐tracking taxa and pathways, particularly *Bacteroides stercoris* and the bacterial secretion‐system pathway, as potential microbial markers for suicide risk in MDD.

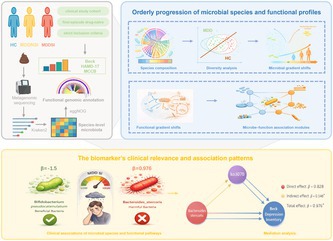

## 1 Introduction

Major depressive disorder (MDD) is a psychiatric condition primarily characterized by persistent low mood and impaired cognitive function, with a global lifetime prevalence of approximately 19% and extremely high disability rates [1, 2]. Moreover, the incidence of MDD continues to rise, with adolescents representing a substantial proportion of patients and contributing to a considerable societal burden [3, 4, 5]. Despite ongoing research advancements, MDD exhibits significant clinical heterogeneity, encompassing multidimensional symptom presentations and complex neurobiological mechanisms [6, 7, 8]. Among these manifestations, suicidal ideation (SI) reflects a particularly severe and complex clinical presentation; compared with MDD without SI (MDDNSI), MDD patients with SI (MDDSI) typically exhibit more complicated symptom profiles and greater functional impairment [9, 10, 11, 12]. However, the biological basis of MDDSI remains poorly understood, highlighting the need to identify specific biomarkers to clarify depression heterogeneity and facilitate personalized treatment.

In recent years, increasing attention has been directed toward the microbiota‐gut‐brain axis (MGBA), emphasizing the crucial role of gut microbiota in regulating host neuropsychiatric health [13, 14, 15, 16, 17, 18]. Numerous studies have reported substantial disruptions in both microbial composition and function in patients with MDD compared with healthy controls (HC) [17, 18, 19, 20, 21, 22]. For example, several studies have reported that *Bacteroides* species such as *Bacteroides stercoris* , *Bacteroides fragilis* , and *Bacteroides eggerthii* are significantly increased in MDD, whereas short‐chain fatty acid (SCFA)‐producing taxa, including *Blautia obeum* and *Blautia wexlerae* , are significantly decreased [23, 24]. In addition, because microbial taxa are intrinsically linked to metabolic capacity, MDD is characterized by marked disruptions across major microbial metabolic pathways, including widespread disturbances in tryptophan and kynurenine metabolism [25, 26].

However, most previous investigations relied on 16S rRNA sequencing, which is limited to genus‐level resolution and therefore unable to capture finer taxonomic and functional details [27, 28]. In contrast, shotgun metagenomic sequencing offers higher‐resolution profiling at the species level and beyond [29]. Despite these observations, existing studies remain highly heterogeneous [30], which may partly result from the clinical heterogeneity of MDD itself. These limitations highlight the need to investigate gut microbiota alterations within more refined clinical subtypes to obtain clearer biological insights.

Research on mental disorders is gradually shifting from traditional single‐diagnosis paradigms toward more refined phenotypic stratification, a trend confirmed across multiple psychiatric conditions [31, 32]. In schizophrenia and bipolar disorder, distinct clinical subtypes such as those associated with body mass index (BMI) have been identified as possessing specific microbial signatures [33, 34]. Similar phenomena have been observed in MDD, where phenotypes such as sex [35], obesity levels [36], and symptom severity influence gut microbial composition [37]. For instance, studies stratifying MDD based on disease severity reveal markedly elevated *Bacteroides* and significantly reduced *Ruminococcus* and *Eubacterium* in moderate‐to‐severe patients, suggesting that refined subtyping aids in identifying more biologically meaningful microbial abnormalities [38].

Notably, microbial differences are even more pronounced in phenotypes closely associated with suicide risk. Previous research reported that individuals with a history of suicide attempts exhibit not only higher microbial richness but also markedly altered β‐diversity patterns, characterized by increased *Fenollaria timonensis* and decreased *Corynebacterium aurimucosum* [39]. Additionally, reduced α‐diversity was observed in those engaging in non‐suicidal self‐injury, with *Mitsukella* emerging as a key microbial marker distinguishing NSSI from participants with depression alone [40]. For MDDSI, studies have also reported positive correlations between certain *Phascolarctobacterium* strains and scores of SI [41]. However, this evidence primarily relies on 16S rRNA sequencing with limited resolution, making it challenging to elucidate deeper microbiological mechanisms. Concurrently, the concept of a disease “continuum” is gaining prominence [42]. The transition from HC‐MDDNSI‐MDDSI reflects a graded increase in clinical severity, yet how the gut microbiota change across this progression remains poorly understood.

To address this critical knowledge gap, we conducted a systematic study in a rigorously characterized cohort of first‐episode, drug‐naïve MDD patients, including 221 participants comprising HC, MDDNSI, and MDDSI matched for sex, age, and BMI. Through shotgun metagenomic sequencing combined with ordinal regression modeling, we identified multiple microbial species and metabolic pathways exhibiting continuous gradient changes along symptom severity, revealing their systematic associations with suicidal ideation, depression symptom severity, and cognitive function. Furthermore, by integrating mediation analysis, we constructed and validated a potential “microbiota‐metabolism‐clinical” pathway, providing a new mechanistic framework for understanding the microbial underpinnings of MDD and its associated suicide risk.

## 2 Materials and Methods

### 2.1 Participant Recruitment

A total of 221 participants were enrolled in this study, comprising 104 first‐episode, drug‐naïve MDD patients and 117 sex‐, age‐, and BMI‐matched HC. All procedures were approved by the Ethics Committee of the Affiliated Brain Hospital of Guangzhou Medical University (Ethics approval ID: (2025) No. 056) and conducted in accordance with the Declaration of Helsinki [43]. Among the MDD patients, 52 presented with SI, while the other 52 had no such symptoms. The diagnosis of MDD was independently confirmed by two trained psychiatrists according to the *Diagnostic and Statistical Manual of Mental Disorders, Fifth Edition* (DSM‐5) [44]. For each participant, fecal sample collection, cognitive function assessments, and clinical information collection were conducted on the same day at the same hospital. The recruitment criteria and procedures were consistent with previous studies [21, 45], and detailed information is provided in the Supporting Information (Note S1).

### 2.2 Clinical Characteristics Collection

Clinical severity in patients with MDD was assessed using the 17‐item Hamilton Depression Rating Scale (HAMD‐17), a widely used instrument that evaluates depressive symptoms across domains, including mood, insomnia, and cognitive and somatic complaints [46]. SI was determined based on item 3 of the HAMD‐17; a score of ≥ 2 was classified as indicative of SI, and a score of < 2 as absent [47]. In addition, the Beck Scale for Suicide Ideation was administered to assess the severity of suicidal ideation, including thoughts, plans, and behaviors within the past week. The total score was recorded for each patient [48]. One of the widely used cognitive function tools is the MATRICS Consensus Cognitive Battery (MCCB), which has been validated in many countries [49, 50]. This study focused on five cognitive domains, including processing speed (SoP), attention and vigilance (AV), working memory (WM), verbal learning and memory (VerLM), and visual learning and memory (VisLM), and derived the corresponding domain‐specific scores from the MCCB.

### 2.3 Fecal Sample Collection and DNA Extraction

**FIGURE 1:**
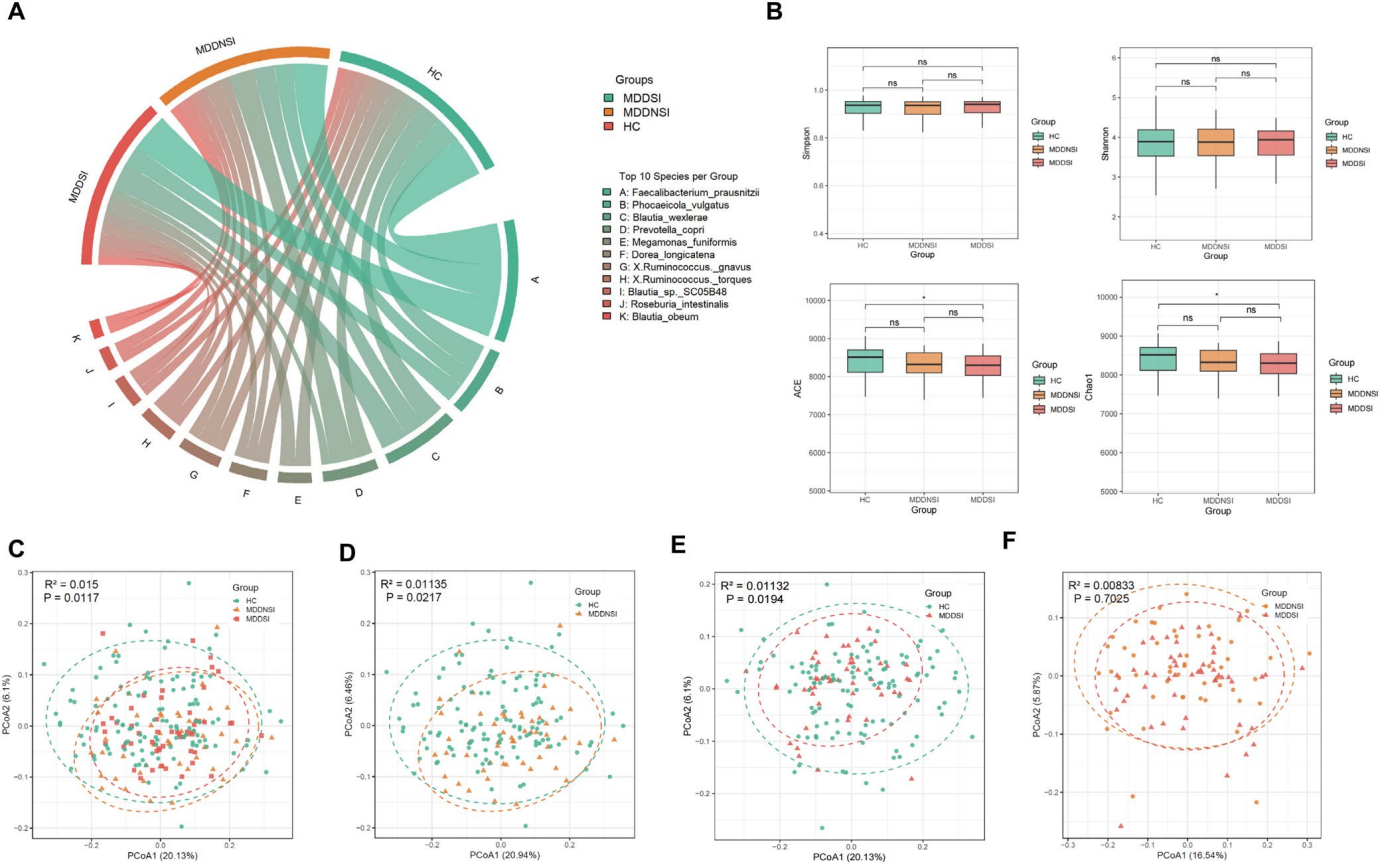
The figure summarizes microbial community features across HC, MDDNSI, and MDDSI groups. (A) The top ten most abundant species identified within each group. (B) α‐diversity indices (Simpson, Shannon, ACE, and Chao1), with asterisks indicating significant group differences. (C–F) β‐diversity of bacterial communities based on Bray–Curtis dissimilarity. Principal coordinates analysis (PCoA) illustrates clustering patterns of bacterial communities across the three groups, with ellipses indicating 95% confidence intervals. Statistical significance (*p*‐value) and effect size (R^2^) of β‐diversity differences were assessed using PERMANOVA (9999 permutations, two‐sided test). (D–F) show pairwise comparisons between groups.

Fresh fecal samples were obtained from each participant, and all of the samples were stored at −80°C until DNA extraction. We collected fecal samples in the morning, and participants were asked not to eat for 12 h before the fecal sample was collected. A total of 200 mg of each fecal sample was used for DNA extraction. Metagenomic libraries were prepared using the NEBNext Ultra II DNA Library Prep Kit for Illumina (New England BioLabs). Whole‐metagenome sequencing was performed on the Illumina NovaSeq 6000 platform with 2 × 150 bp paired‐end reads. To rigorously control for technical variation, all metagenomic libraries were prepared and sequenced together in a single run on the Illumina NovaSeq 6000 platform. Samples from different clinical groups were fully randomized during library preparation within this run. The clear separation by clinical group in the PCoA plot (Figure 1C), with no visual evidence of sub‐clustering, indicates that biological differences dominate over any potential minor technical variation.

### 2.4 Metagenomic Sequence Analysis

Raw metagenomic sequencing data were processed using fastp [51] and kneaddata (https://github.com/biobakery/kneaddata). First, adapter sequences and low‐quality bases were trimmed using fastp [51]. Then, host DNA contamination was removed by aligning reads to the host reference genome using kneaddata, and only clean reads were retained for downstream analysis. Taxonomic profiling was performed using Kraken2 [52] and Bracken to estimate the gut microbiota abundance at the species level. Default parameters were applied, and a standard reference database was used for classification. Clean reads were assembled into contigs using MEGAHIT (version 1.2.9) with default parameters [53]. Contigs longer than 1,000 bp were retained using seqtk (version 1.4; https://github.com/lh3/seqtk). The abundance of contigs was quantified using Salmon (version 1.10.3), which employed a pseudo‐alignment approach against sample‐specific contig indices [54]. Functional annotation of the assembled contigs was conducted using EggNOG‐mapper with default parameters [55]. This provided annotations across multiple databases, including Kyoto Encyclopedia of Genes and Genomes (KEGG) pathways, enabling downstream functional analyses [56].

### 2.5 Bioinformatics Analysis

We performed α‐ and β‐diversity analyses on all samples to characterize the overall structure of the gut microbiota. α‐diversity was used to quantify within‐sample microbial diversity, using the Shannon, Simpson, Chao1, and ACE indices to capture species richness and evenness. β‐diversity was used to assess between‐sample differences in microbial composition. Bray−Curtis distance matrices were computed from species abundance profiles and visualized using principal coordinate analysis (PCoA) in the R “vegan” package (version 4.0.2). Intergroup differences in β‐diversity were evaluated using a permutation‐based multivariate analysis of variance (PERMANOVA), with 9999 random permutations used to assess overall community‐structure separation across groups. Hierarchical All‐against‐All Association (HALLa) was applied to characterize interaction patterns between gut microbiota and putative metabolic pathways. This framework combines Spearman correlation, hierarchical clustering, and multiple testing correction, while accounting for feature co‐occurrence, to robustly identify significant bacterial species‐metabolic pathway association modules (FDR‐adjusted *p* < 0.05).

### 2.6 Statistical Analyses

Differences between categorical variable groups were assessed using chi‐square tests; normality of continuous variables was evaluated via Shapiro–Wilk tests prior to analysis. For normally distributed data, independent samples *t*‐tests were used for two‐group comparisons, while one‐way ANOVA was employed for three or more groups. For non‐normally distributed data, Mann–Whitney U tests were used for two groups, and Kruskal−Wallis tests for three or more groups. A post hoc power analysis was conducted using G*Power 3.1 software based on the observed effect size (Cohen's d) for key significant findings. For instance, for the comparison of *Bacteroides stercoris* abundance between the MDDSI and HC groups, the achieved power was 31.6% (α = 0.05, two‐tailed, d = 0.248).

To explore trends in gut microbial species and KEGG pathway abundance across disease severity, participants were coded as HC, MDDNSI, and MDDSI (0, 1, 2). Ordered logistic regression models were constructed for each species and pathway to extract β coefficients and corresponding *p*‐values. Subsequently, linear regression assessed associations between gut microbial features, KEGG pathway abundance, and clinical scales (HAMD‐17, Beck, MCCB). To assess the specificity of microbial associations to suicidal ideation independent of overall depression severity, a sensitivity analysis was performed. Significant features were re‐evaluated using linear regression, with the HAMD‐17 total score included as a covariate. The proportional odds assumption for the ordinal logistic regression models was assessed using the Brant test. All species and pathways reported as significant in the main analysis satisfied this assumption (Brant test, *p* > 0.05). Mediation analysis was performed using Python (Pingouin 0.3.12), with differential species as independent variables, differential KEGG pathways as mediators, and clinical scales as dependent variables. Throughout all analyses, sex, age, BMI, and educational years were consistently controlled to minimize covariate effects on microbial abundance and clinical indicators. All statistical tests underwent FDR correction using the Benjamini−Hochberg method, with significance set at FDR‐adjusted *p* < 0.05.

## 3 Results

### 3.1 Clinical and Demographic Characteristics

A total of 52 MDDSI, 52 MDDNSI, and 117 HC subjects matched for age, sex, and BMI were enrolled in this study. The MDDSI group showed significantly higher Beck scores compared with the MDDNSI group and also displayed significantly elevated HAMD scores (Table 1). These findings indicate that patients with SI exhibit significantly more severe depressive symptoms compared with those without SI. In addition, patients showed significantly lower cognitive performance than HC in multiple domains, including SoP, AV, WM, VerLM, and VisLM, suggesting overall cognitive impairment in the MDD population.

**TABLE 1:** Demographic and clinical characteristics between MDDSI, MDDNSI, and HC.

	MDDSI (*n* = 52)	MDDNSI (*n* = 52)	HC (*n* = 117)	*p*
Sex (Male: Female)	15:37	21:31	50:67	0.227
Age	22.83 ± 4.65	24.15 ± 4.71	24.34 ± 4.92	0.16
BMI	20.58 ± 4.23	20.73 ± 3.37	21.57 ± 3.46	0.185
Educational years	14.29 ± 2.46	14.50 ± 2.68	16.18 ± 2.19	< 0.001
HAMD	24.75 ± 4.45	20.63 ± 4.50	—	< 0.001
Beck	36.34 ± 10.02	31.56 ± 9.39	—	< 0.050
SoP	31.69 ± 10.00	34.96 ± 9.49	46.94 ± 9.68	< 0.001
AV	32.08 ± 11.82	34.40 ± 11.00	43.44 ± 9.35	< 0.001
WM	40.88 ± 11.66	39.10 ± 12.59	46.91 ± 10.11	< 0.001
VerLM	33.31 ± 10.67	35.08 ± 9.41	41.24 ± 8.91	< 0.001
VisLM	41.33 ± 8.05	40.19 ± 7.65	45.79 ± 9.08	< 0.001

Abbreviations: MDD, major depressive disorder; MDDSI, major depressive disorder with suicidal ideation; MDDNSI, major depressive disorder without suicidal ideation; BMI, body mass index; SoP, Speed of processing; WM, working memory; VerLM, verbal learning and memory; VisLM, visual learning and memory.

### 3.2 Gut Microbial Composition and Diversity Analysis

To investigate dominant bacterial species across different ecological niches, we compared the gut microbiota composition of the three groups. We found that the majority of dominant taxa were shared among HC, MDDSI, and MDDNSI. For example, *Faecalibacterium prausnitzii* [57], a well‐recognized beneficial and health‐associated taxon, was the highest‐abundance species in all groups. Interestingly, the butyrate‐producing species *Blautia obeum* was dominant only in patient groups (Figure 1A). In addition, α‐diversity analysis showed that only the MDDSI group had significantly lower ACE and Chao1 indices compared with HC, indicating reduced richness and rare species abundance. However, no significant differences were observed among the three groups in evenness‐based indices, including Simpson and Shannon (Figure 1B). β‐diversity analysis based on Bray−Curtis distances revealed significant overall differences among the three groups, and post hoc pairwise comparisons further showed that both MDDNSI and MDDSI groups differed significantly from HC (Figure 1C–E). This indicates that the gut microbial communities of MDD patients deviate substantially from the healthy baseline. However, no significant differences were observed between MDDSI and MDDNSI (Figure 1F), suggesting that while both represent depressive states, the overall community‐level microbial structures remain largely similar, and more refined analyses at the species and functional levels are required to identify disease‐specific microbial alterations.

### 3.3 Disease‐Specific Microbial Signatures of MDD

**FIGURE 2:**
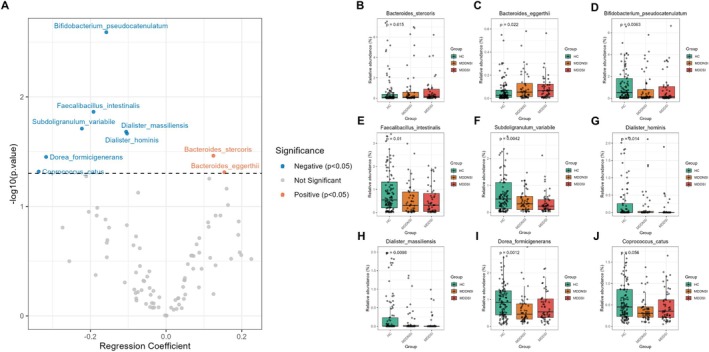
Severity‐associated microbial taxa along the HC‐MDDNSI‐MDDSI continuum. (A) Volcano plot showing taxa significantly associated with disease severity, identified by ordinal logistic regression. Positive coefficients indicate progressive enrichment, whereas negative coefficients indicate depletion across the spectrum. (B–J) Relative abundances of representative severity‐associated taxa across the three clinical states.

Disease severity was modeled on a continuous axis from HC to MDDNSI to MDDSI using ordinal logistic regression, identifying 9 taxa significantly associated with disease progression (Figure 2A). Among these, putative pathogenic species *Bacteroides stercoris* and *Bacteroides eggerthii* showed a stepwise increase in abundance (Figure 2B,C), consistent with previous reports of enrichment in different disorders [58]. In contrast, seven beneficial taxa, including *Bifidobacterium pseudocatenulatum* , *Faecalibacillus intestinalis*, *Subdoligranulum variabile* , *Dialister massiliensis*, *Dialister hominis*, *Dorea formicigenerans* , and *Coprococcus catus* , most of which are SCFA‐producing bacteria, showed a continuous decline along the HC to MDDNSI to MDDSI spectrum [59, 60] (Figure 2D–J). It is important to note that the significant association for these species, as identified by ordinal regression, reflects a progressive trend across the severity continuum, which is the primary analysis for this hypothesis and may not always correspond to significant differences in all pairwise group comparisons. The sensitivity analysis results remained stable after controlling for depression severity (Figure S1A). Briefly, the association of features such as *Bacteroides stercoris* with the SI group appeared to be robust even after this adjustment.

### 3.4 Disease‐Specific Potential Functional Pathways

**FIGURE 3:**
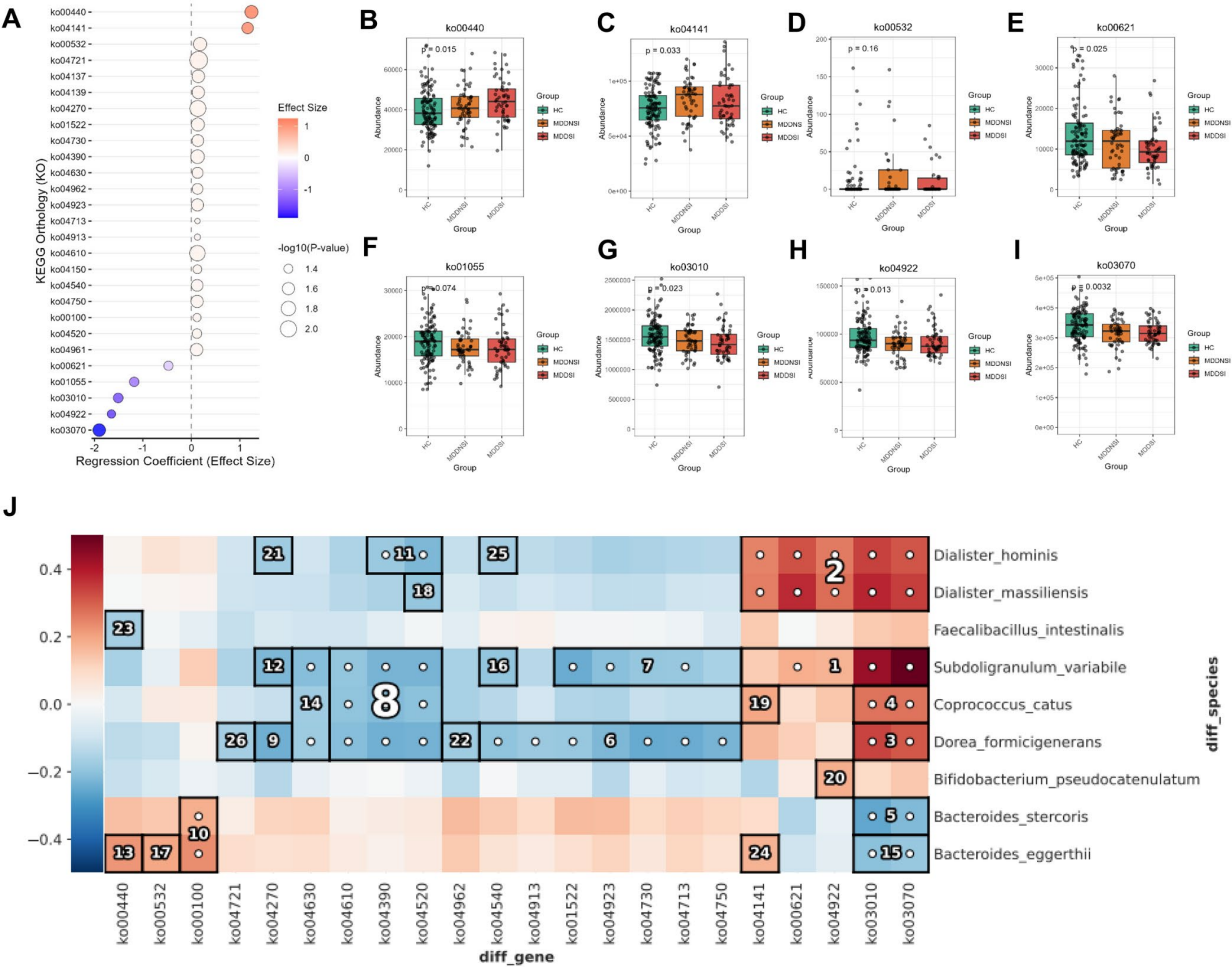
Severity‐linked KEGG pathways and microbe‐pathway relationships. (A) KEGG metabolic pathways significantly associated with disease severity. (B–I) Relative abundances of representative pathways across HC, MDDNSI, and MDDSI groups. (J) Correlation heatmap linking severity‐associated taxa with pathways; red indicates positive and blue indicates negative correlations, with values reflecting effect size and significance. Asterisks (*) indicate statistically significant correlations after multiple testing correction (FDR‐adjusted *p* < 0.05).

KEGG pathway analysis identified 27 significantly altered microbial metabolic pathways based on ordinal logistic regression (Figure 3A). Pathways that progressively decreased from HC to MDDSI included Bacterial secretion system (ko03070), Glucagon signaling pathway (ko04922), Ribosome (ko03010), Biosynthesis of vancomycin‐group antibiotics (ko01055), and Dioxin degradation (ko00621) (Figure S2A–S, Figure S1B). In contrast, pathways such as Phosphonate and phosphinate metabolism (ko00440), Protein processing in endoplasmic reticulum (ko04141), Glycosaminoglycan biosynthesis‐chondroitin sulfate/dermatan sulfate (ko00532), and Synaptic vesicle cycle (ko04721) increased with disease severity (Figure 3B–I).

### 3.5 Integrated Microbe‐Function Association Analysis

HALLA analysis revealed coordinated clusters of microbial taxa and metabolic pathways. Disease‐increasing taxa, such as *Bacteroides stercoris* and *Bacteroides eggerthii* , act as a coordinated module rather than associating with individual pathways, forming structured associations with multiple metabolic pathways, indicative of a microbe‐function coupling pattern. Conversely, the beneficial taxon *Bifidobacterium pseudocatenulatum* was associated with the disease‐decreasing pathway ko04922 (Figure 3J). These findings reveal specific microbe‐function synergistic variation modules that not only support the concept of gut ecosystem functional remodeling during MDD progression but also suggest these modules may respectively participate in the exacerbation or remission processes of MDD.

### 3.6 Associations With Clinical Characteristics

**FIGURE 4:**
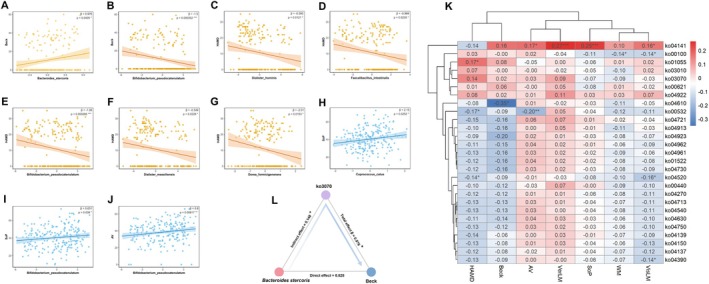
Clinical relevance and mediation effects of severity‐associated microbial features. (A, B) Associations between severity‐associated taxa and Beck scores. (C–G) Associations with total HAMD scores. (H–J) Associations with cognitive performance (SoP and AV). (K) Heatmap of correlations between severity‐linked pathways and clinical measures. Asterisks (*) indicate statistically significant correlations after multiple testing correction (FDR‐adjusted *p* < 0.05). (L) Mediation analysis showing that the effect of *Bacteroides stercoris* on Beck scores is partially mediated through the bacterial secretion system pathway.

*Bacteroides stercoris* was positively correlated with Beck scores (Figure 4A), while *Bifidobacterium pseudocatenulatum* was negatively correlated (Figure 4B), suggesting opposing effects. Several decreasing taxa, including *Dialister hominis*, *Faecalibacillus intestinalis*, *Dialister massiliensis*, *Bifidobacterium pseudocatenulatum* , and *Dorea formicigenerans* , showed significant negative associations with HAMD total scores (Figure 4C–G), indicating potential protective roles. Notably, these taxa with reduced relative abundance were also significantly associated with cognitive decline, exhibiting a clear and consistent pattern. *Bifidobacterium pseudocatenulatum* showed positive correlations with AV and SoP (Figure 4I,J), and *Coprococcus catus* correlated positively with SoP (Figure 4H). Functionally, ko04141 correlated positively with multiple cognitive domains, whereas ko00100, ko04520, ko04390, and ko00532 correlated negatively. Additionally, ko01055 correlated positively with HAMD, whereas ko04610 correlated negatively with Beck scores (Figure 4K).

### 3.7 Mediation Analysis: Microbiome‐Function‐SI

Given that *Bacteroides stercoris* increased with disease severity, correlated positively with Beck scores, and was associated with pathway ko03070, mediation analysis was performed (Figure S3). Results showed a significant direct effect on Beck score (*β* = 0.828) and a total effect of *β* = 0.976 via the *Bacteroides stercoris‐*ko03070‐Beck score pathway, with an indirect effect of 0.148, indicating partial mediation (Figure 4L). This suggests that *Bacteroides stercoris* may contribute to SI partly through the modulation of ko03070. However, although *Bifidobacterium pseudocatenulatum* was significantly associated with Beck scores, no specific mediating pathway was identified.

## 4 Discussion

To the best of our knowledge, this study established the first continuous clinical severity spectrum spanning HC, MDDNSI, and MDDSI, revealing systematic changes in gut microbial structure and function along an increasing suicidal risk gradient. Notably, *Bacteroides stercoris* progressively increased with disease progression, while *Faecalibacillus intestinalis* continuously decreased. Concurrently, key metabolic pathways such as the bacterial secretion system weakened with increasing severity, forming stable bacteria‐function interaction modules with differentially abundant microbial communities. Further analysis indicated a significant association between *Bacteroides stercoris* and SI, with the bacterial secretion system acting as a potential intermediary mechanism.

Although HC, MDDNSI, and MDDSI show a clear gradient of clinical severity [41], characterized by progressively intensified depressive symptoms accompanied by cognitive decline, the overall structural differences in the gut microbiome remain relatively subtle. α‐diversity analysis reveals significant alterations in ACE and Chao1 indices compared to HC only in MDDSI, indicating that intraspecific ecological redundancy and stability may begin to unravel during more severe symptom stages. Consistent with previous studies, MDDNSI, as a relatively mild stage, does not yet show noticeable changes in intraspecific diversity [38]. Conversely, β‐diversity analysis reveals that both MDDNSI and MDDSI have markedly diverged from HC, yet no significant differences exist between them. This indicates that the unique clinical feature of SI remains difficult to capture when assessed solely through overall community structure distance. Under disease conditions, the microbiome may become increasingly heterogeneous and unstable [61], which may explain why similarity‐based distance metrics often fail to identify distinct microbial patterns linked to specific clinical manifestations. This may partly explain why previous studies frequently identified differences between MDD and HC but struggled to capture microbial signals associated with specific clinical phenotypes [24].

At the species level, the stepwise shift in microbial patterns identified along the HC‐MDDNSI‐MDDSI continuum in this study aligns strongly with previously reported specific microbial signatures of MDD [23]. On the one hand, *Bacteroides stercoris* and *Bacteroides eggerthii* , which progressively increase with symptom severity, have been identified as potential pathogenic bacteria primarily observed in moderate‐to‐severe MDD but not prominent in mild cases [38]. These microbial communities are often associated with enhanced lipopolysaccharide synthesis and elevated pro‐inflammatory factors [62, 63]. Previous studies suggest that MDD is characterized by increased intestinal permeability, low‐grade systemic inflammation, and microbe‐driven immune dysregulation, with certain taxa potentially amplifying host inflammatory responses [64, 65]. The progressive increase of these taxa across the clinical severity spectrum suggests that the gut ecosystem of MDDSI patients may gradually shift toward a state more prone to immune activation and neuroinflammation.

Conversely, several microbial species whose abundance declined with increasing disease severity belong to short‐chain fatty acid (SCFA) producing bacteria [64, 65, 66, 67, 68, 69, 70]. SCFAs, particularly butyrate‐producing bacteria, are key metabolic substances that play vital roles in maintaining intestinal barrier integrity, suppressing inflammatory pathways, and regulating brain function [71, 72]. Their progressive reduction indicates that the body's anti‐inflammatory metabolic capacity progressively weakens as the disease worsens. Overall, the HC‐MDDNSI‐MDDSI gradient reflects disease‐specific microecological shifts shaped by heightened proinflammatory activity alongside diminished anti‐inflammatory capacity, consistent with the emerging concept of microbial functional balance [73]. Future efforts should focus on developing strategies to intervene in microbial community equilibrium and achieve personalized, precise regulation.

At the functional level, microbial metabolic pathways also undergo changes during disease progression. Similar to microbial alterations, pathways that intensify with increasing severity involve multi‐level processes such as phosphonate and phosphinate metabolism, protein processing in the endoplasmic reticulum, and the synaptic vesicle cycle. These functions have previously been closely associated with metabolic stress, host cellular stress responses, and neural activity regulation [74, 75]. In contrast, pathways that progressively diminish with disease severity primarily involve core biosynthetic processes such as the bacterial secretion system, ribosome function, and biosynthesis of vancomycin‐group antibiotics. These findings are consistent with microbial dysregulation patterns reported in animal models of depression, where alterations in bacterial secretion systems and ribosomal functions have been associated with synaptic dysfunction in chronic stress‐related affective disorders [76, 77].

Collectively, the functional pattern of “elevated oxidative stress coupled with restricted biosynthesis” suggests that gut microbiota may provide an important functional background for differentiating disease severity by influencing host energy homeostasis and neurosignaling pathways. Furthermore, this study observed highly synergistic and cluster‐associated structures between differential microbial communities and functional pathways, indicating that disease states may exert targeted effects on specific “microbiota‐function” modules [78].

In terms of clinical relevance, this study identified several potentially beneficial bacteria whose abundance decreased along the disease continuum and showed negative correlations with depression severity while being positively associated with cognitive function. Among these, *Bifidobacterium pseudocatenulatum* ‐related taxa were negatively associated with HAMD total scores and SI and positively associated with cognitive function [79]. Given its known roles in SCFA production and bile acid metabolism regulation, this pattern suggests it may exert holistic neuroprotective effects across both symptom and cognitive dimensions [59, 80].

In addition, we observed for the first time that the abundance of the complement and coagulation cascade pathway (ko04610) progressively increased with disease severity. This finding aligns strongly with recent proposals that dysregulation of the complement‐coagulation axis may constitute an early pathophysiological mechanism in psychiatric disorders. Longitudinal studies indicate that abnormal changes in complement and coagulation‐related proteins can precede the onset of psychiatric symptoms by several years, suggesting the potential for this system to serve as a “prodromal” biomarker [81]. Previous research has also indicated that suicidal behavior in MDD patients correlates with distinct immune‐coagulation profiles, with “excessive activation of the extrinsic coagulation pathway” regarded as a key biological process in suicide risk [82]. However, in contrast to these findings, our study observed a significant negative correlation between this pathway and suicidal ideation. Future studies with more refined designs are needed to further elucidate the relationship between this metabolic pathway and suicidal ideation.

More importantly, SI may be driven by multiple biological pathways. The effects of *Bacteroides stercoris* are partially mediated by the bacterial secretion system pathway, which is closely associated with the release of inflammation‐related molecules [83], consistent with previous evidence linking inflammation to mood and stress regulation [84]. In contrast, *Bifidobacterium pseudocatenulatum* , a prototypical homeostatic probiotic, showed a negative correlation with SI but did not exhibit clear single‐pathway mediation. This may reflect its effects relying more on multidimensional, low‐intensity regulatory mechanisms such as SCFAs, barrier stabilization, or immune tolerance, which extend beyond the scope of current KEGG pathway analysis. Collectively, these findings provide valuable insights for future targeted interventions based on *Bacteroides stercoris* and potential protective applications of *Bifidobacterium pseudocatenulatum* [85].

Our functional analysis revealed a significant alteration in microbial KEGG pathways, most notably a reduction in the bacterial secretion system (ko03070). This finding invites mechanistic speculation on its potential implications for host physiology. The bacterial secretion system is a fundamental mechanism for microbiota‐host interaction, enabling the transport of proteins and other molecules. A putative decrease in its capacity could have several implications relevant to depression and SI. For instance, an impaired secretion system may compromise microbial interactions with the intestinal epithelium, potentially affecting gut barrier integrity. This could, in turn, facilitate the translocation of microbial products, contributing to the systemic inflammatory state often observed in severe MDD [86]. Furthermore, diminished secretion of microbial metabolites or immunomodulatory signals could alter host immune homeostasis and disrupt the production of neuroactive compounds (e.g., those derived from tryptophan), thereby influencing gut‐brain communication [87]. Thus, the observed functional shift may represent a mechanistic link between the taxonomic changes and the inflammatory and metabolic disturbances characteristic of severe MDD with SI.

Despite rigorous measures taken in cohort construction and confounding factor control, this study has several limitations. First, the sample size, particularly for the MDDSI group (*n* = 52), was limited by the challenge of recruiting first‐episode drug‐naïve patients with active suicidal ideation. A post hoc power analysis confirmed that this sample size provided limited statistical power to detect small effect sizes. Therefore, while the positive findings are robust, negative results (e.g., the lack of significant β‐diversity between MDDSI and MDDNSI) should be interpreted with caution. Future validation in larger, multi‐center cohorts is essential to confirm these findings and enhance the generalizability of the conclusions. At the same time, a limitation of this study is that the classification of suicidal ideation was based solely on item 3 of the HAMD‐17. While this is a common screening measure in depression studies, it is not as nuanced as dedicated scales (e.g., the C‐SSRS). Therefore, our findings should be interpreted as an initial exploration within the HAMD‐17 framework, and future validation with more specific instruments is warranted. Second, the cross‐sectional design precludes revealing the dynamic changes in SI; prospective follow‐up studies will be conducted to further determine its evolutionary trajectory. Third, key confounders such as detailed dietary habits, physical activity, smoking, and antibiotic use were not controlled for. Their absence limits our ability to definitively attribute the microbial changes solely to MDD/SI. Future studies should prioritize collecting these data to isolate the specific effects of the disorder. Finally, while metagenomic sequencing can analyze microbial composition and potential functions, it cannot encompass multi‐level biological processes such as metabolism and immunity. Integrating multi‐omics approaches in future studies will facilitate a more comprehensive understanding of the underlying mechanisms [88, 89, 90, 91].

## 5 Conclusion

In conclusion, this study delineates the stepwise changes in gut microbial composition and functional pathways along the disease severity continuum from healthy controls to MDD patients with or without SI. It further reveals significant associations between these changes and depressive symptoms, SI, and cognitive performance. Mediation analysis further identified a microbial functional axis comprising *Bacteroides stercoris* , bacterial secretion system pathways, and Beck scores. These findings provide clear features for understanding the microbiome gradient underlying clinical severity and offer new perspectives for future clinical subtyping and precision intervention studies.

## Author Contributions

Liqin Liang: Conceptualization, Data curation, Methodology, Formal analysis, Validation, Visualization, Writing – original draft. Shuhao Chen: Methodology, Writing – review and editing. Baoyuan Zhu: Methodology, Writing – review and editing. Xiaodan Lu: Methodology, Writing – review and editing. Yuanyuan Huang: Resources, Methodology, Writing – review and editing. Shuhong Wang: Methodology, Writing – review and editing. Wei Wang: Writing – review and editing. Rui Han: Writing – review and editing. Jing Zhou: Methodology, Writing – review and editing. Dongsheng Xiong: Methodology, Writing – review and editing. Hehua Li: Methodology, Writing – review and editing. Xiaobo Li: Methodology, Writing – review and editing. Yuping Ning: Methodology, Writing – review and editing. Fengchun Wu: Resources, Supervision, Writing – review and editing. Kai Wu: Conceptualization, Funding acquisition, Resources, Supervision, Writing – review and editing. All authors read and approved the final manuscript.

## Funding

This work was supported by the National Key Research and Development Program of China (2023YFC2414500, 2023YFC2414504, 2025YFC3410000, and 2025YFC3410005), the National Natural Science Foundation of China (82271953 and 82301688), the Key Research and Development Program of Guangdong (2023B0303020001 and 2023B0303010003), the Natural Science Foundation of Guangdong Province (2024A1515013058, 2025A1515010507, and 2023A1515011383), the Guangdong Key Laboratory of Battery Safety at Guangzhou Institute of Energy Testing (2019B121203008‐KJ‐2024‐040/KJ‐2024‐041), the Science and Technology Program of Guangzhou (2025A03J3357), the Clinical Collaboration Project on Integrated Traditional Chinese and Western Medicine for Major and Difficult Diseases (Bipolar Disorder, ZDYN‐2024‐A‐121), the Research Capacity Improvement Project of Guangzhou Medical University (2024SRP200), Guangzhou Key Clinical Specialty (Clinical Medical Research Institute), the Announcement and Leading Science and Technical Foundation of Guangzhou Civil Affairs (GCAAL2022001), and the Guangzhou Planned Project of Science and Technology (2023B04J0106 and 2025B04J0011).

## Ethics Statement

The study protocol was approved by the Ethics Committee of the Affiliated Brain Hospital of Guangzhou Medical University (Ethics approval ID: (2025) No. 056) and was conducted in accordance with the Declaration of Helsinki.

## Consent

All participants provided written consent for the publication of anonymized data in scientific journals.

## Conflicts of Interest

The authors declare no conflicts of interest.

## Supporting information


**Note S1:** Participant Recruitment Criteria.
**Figure S1:** Microbial taxa and KEGG pathways associated with disease severity across clinical groups.
**Figure S2:** Relative abundances of differential KEGG pathways across the HC‐MDDNSI‐MDDSI continuum.
**Figure S3:** Standard three‐variable path model of mediation analysis.

## Data Availability

Raw metagenomic sequencing data are not publicly available due to patient privacy restrictions and ongoing related studies but may be requested from the corresponding author under a Data Transfer Agreement (DUA) outlining ethical use terms. The analysis code generated in this study are openly available in the GitHub repository at: https://github.com/LQL‐O/HC_MDDNSI_MDDSI.
