# Supplementary material for: Continuous Alterations in the Gut Microbial Landscape Associated With Suicidal Ideation in First‐Episode Drug‐naïve Major Depressive Disorder
Source: CNS Neurosci Ther. 2026 Jun 18;32(6):e70892. doi: 10.1002/cns.70892 (PMC13279886; doi:10.1002/cns.70892)
Supplement: Supplementary file 1 — Note S1: Participant Recruitment Criteria. Figure S1: Microbial taxa and KEGG pathways associated with disease severity across clinical groups. Figure S2: Relative abundances of differential KEGG pathways across the HC‐MDDNSI‐MDDSI continuum. Figure S3: Standard three‐variable path model of mediation analysis. [file CNS-32-e70892-s001.docx]

**Supporting information**

**Note S1. Participant Recruitment Criteria**

All patients with MDD met the following inclusion criteria: (1) patients with depression meeting the DSM-5 diagnostic classification criteria for major depressive disorder; (2) first-episode depression with a disease course not exceeding 2 years; (3) no history of psychiatric drug use; and (4) age between 18 and 45 years. The exclusion criteria were as follows: (1) presence of serious physical diseases; (2) diagnosis of other mental illnesses or organic brain dysfunction; (3) history of alcohol or other psychoactive substance abuse; (4) previous head trauma with loss of consciousness or serious sequelae; (5) pregnancy or lactation; (6) history of electroconvulsive therapy; and (7) inability to cooperate with cognitive testing. The research adhered strictly to the Declaration of Helsinki and was conducted under the approval of the ethics committee of the Affiliated Brain Hospital of Guangzhou Medical University.


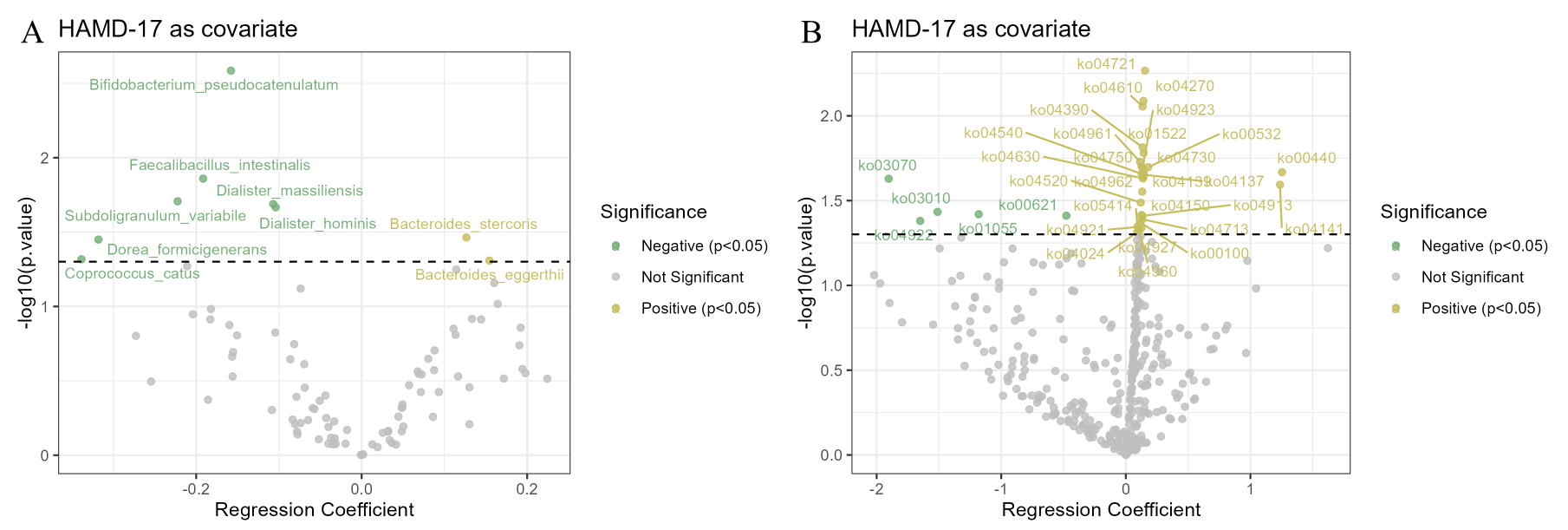


**Figure S1. Microbial taxa and KEGG pathways associated with disease severity across clinical groups.**

(A) Volcano plot showing taxa significantly associated with disease severity, identified by ordinal logistic regression with HAMD-17 total score included as a covariate. (B) Volcano plot showing KEGG pathways identified using the same model.


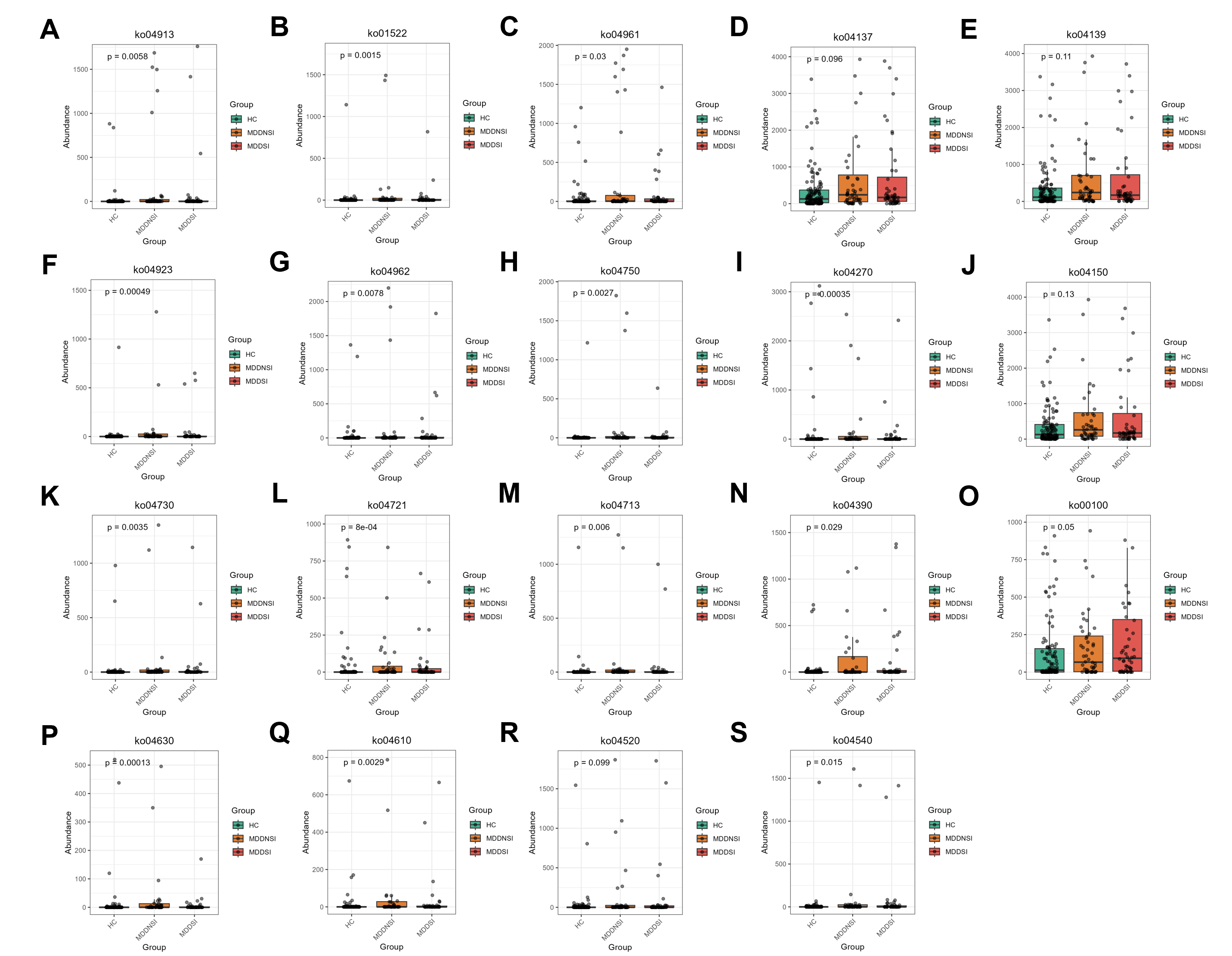


**Figure S2. Relative abundances of differential KEGG pathways across the HC-MDDNSI-MDDSI continuum.**

The KEGG pathways show severity-aligned, continuous shifts across HC, MDDNSI, and MDDSI, reflecting progressive functional remodeling with increasing clinical severity.


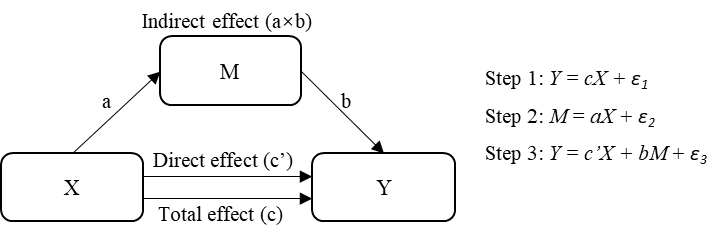


**Figure S3. Standard three-variable path model of mediation analysis.**

The effect of X on Y consists of both a direct path and an indirect path mediated through M.
